# The Effects of Continual Consumption of *Origanum vulgare* on Liver Transcriptomics

**DOI:** 10.3390/ani11020398

**Published:** 2021-02-04

**Authors:** Yadav S. Bajagai, Anita Radovanovic, Jason C. Steel, Dragana Stanley

**Affiliations:** 1 Institute for Future Farming Systems, Central Queensland University, Rockhampton, QLD 4702, Australia; y.sharmabajagai@cqu.edu.au (Y.S.B.); j.steel@cqu.edu.au (J.C.S.); 2 Faculty of Veterinary Medicine, University of Belgrade, 11000 Belgrade, Serbia; anita@vet.bg.ac.rs

**Keywords:** liver, RNAseq, oregano, broiler, steroid hormones, cancer

## Abstract

**Simple Summary:**

The use of phytogenic products has entered mainstream use in the livestock industry as an antibiotic alternative. These products, often based on herbs and spices with established antimicrobial properties, are generally considered as safe and natural, however, they are often administered in high doses and frequency. The direct effects of these products on the livestock animals remains under-reported. Using a transcriptomics, we show that supplementing 2% oregano in feed has direct effects on gene expression in the livers of broilers with a potential range of beneficial and negative side effects.

**Abstract:**

Pathogen control is re-emerging as a significant challenge to the health of both humans and animals. The livestock industry is in the process of massively replacing in-feed antibiotics with organic production friendly plant-based products. Nutrigenomics as a science of the effects of food constituents on gene expression is shedding more light on both benefits and detrimental side-effects of feed additive prolonged consumption on the host, indicating the need to understand the feed-host interactions and their influence on the host disease profile. In this study, we investigated the effects of 2% oregano powder supplementation on the liver gene expression in healthy male broilers from the hatch to 6 weeks of age. Deep RNAseq was performed on average 113.3 million paired and quality trimmed sequences per sample and four samples for the control and treatment each. The results demonstrate the severity of oregano effect on liver gene expression with substantial modifications in steroid hormone regulation, fat and carbohydrate metabolism alterations and strong influence on the host disease and function profile. Oregano supplementation was able to interfere with the transcriptional effects of a range of registered drugs and to significantly transcriptionally inhibit a range of cancer disease categories including liver cancer, and to modify fat and carbohydrate metabolism.

## 1. Introduction

Antibiotic growth promoters (AGPs) are used in the livestock industry to prevent both host and zoonotic disease, control microbial populations and promote the growth of animals [1]. Most economically significant diseases controlled by AGPs are necrotic enteritis (NE) and *Salmonella* [2]. The term “growth promotion” by antibiotic addition refers to increased animal growth in terms of weight gain on the same amount of feed consumed [3], and it is often misleadingly confused in the media as growth hormone supplementation. 

The growth-promoting action of supplemented AGPs to livestock feed is attributed to the reduction of the immune response caused by lack of pathogen pressure. The meat of animals fed AGPs has a higher protein and lower fat percentage [3]. In the industry characterized with high pathogen load and high stocking density, pathogen control in combination with good biosecurity lowered morbidity, morality and inflammation and increased farm productivity [3]. 

Recently, concerns about livestock industry harboring antimicrobial resistance genes that can be transferred to humans resulted in the trend of banning the AGPs as livestock supplement worldwide. Rise in the antimicrobial resistance (AMR) is a process where bacteria are becoming resistant to antibiotics expected to kill them. AMR is now recognized by the World Health Organization (WHO) as a priority research area with dire predictions that the death toll of AMR could be one person every three seconds, amounting to 10 million per year by 2050 and costing the world over USD 100 trillion [4]. 

Initially, the ban of AGP use was perceived as a total disaster by the shell-shocked livestock industry as the disease soared on the farms in the countries that banned AGPs first [5]. Nevertheless, the industry quickly adjusted and made a move towards alternative natural pathogen controlling agents like well-known antimicrobial herbs and spices such as oregano, thyme, garlic, capsicum, cinnamon, and others [6]. Initially, this started as a raw spice or essential oil supplementation, but over the last decade, it evolved to the use of products with controlled doses of the most active antimicrobial components from the herbs and spice origin [7]. Regardless of the large-scale use by industry, very little is known about how products with such a complex mode of action affect different organs and physiological systems as well as general animal health and wellbeing. 

The *Lamiaceae* family, including mint, sage, thyme, marjoram, and oregano, are one of the most widely used natural products for their potent and efficient antimicrobial characteristics. *Origanum vulgare* (oregano) has been widely studied as a phytoadditive. Oregano essential oil generally contains monoterpenes carvacrol and thymol and their biosynthetic precursors *p*-cymene and *y*-terpinene have been shown to be the dominant components [8,9]. Carvacrol and thymol, separately, have antimicrobial effects against bacteria and fungi and when used together, show an additive effect against pathogens [10]. Oregano and its active components are among the most highly used in the poultry industry for pathogen control due to excellent antimicrobial action against major economically important poultry pathogens [7].

Nutritional genomics, also called nutrigenomics, is a rapidly emerging area of science investigating the effects of food formulations or individual food constituents on gene expression and host health [11]. Nutrigenomics opened the horizons for the use of personalized nutrition in patient recovery, disease prevention and treatment. Personalized nutrition in athletes can help optimize body composition and athletic performance [12]. In veterinary nutrition, welfare and performance of farmed, wild and pet animals can benefit from nutrigenomic studies. Many diets and food constituents are holding information on their potential use in health and wellbeing as well as possible adverse effects they may have on the host. At the moment, the high sequencing cost is the primary limiting factor for the projected immense growth and applications of nutrigenomics. 

Presently there is only one transcriptomic study on the effects of oregano supplementation [13], curiously in broiler chicken liver gene expression. Sabino et al. [13] reported inhibition of fatty acid metabolism and insulin signaling pathways in broilers supplemented with oregano extract. They suggested that oregano plays a role in reducing abdominal and visceral fat deposition and that dietary oregano could present an intervention opportunity in obesity and diabetes. Others have also investigated the effects of oregano and other herbal extracts on gene expression using qPCR or ELISA to gain the insight to change of selected target genes [14,15,16]. We hypothesized that high level of continual oregano consumption would have a strong influence on liver gene expression that would exceed previously reported alterations due to cumulative effects of prolonged use, and would likely alter bird predisposition to different types of disease. 

In this manuscript, we present the transcriptional effects of prolonged oregano use on the gene expression in the liver of broiler chickens fed powdered oregano from the hatch to 6 weeks of age. Our results indicate complex interactions of oregano components with the host transcriptional regulation, androgen hormones, cancer-related disease, fat and carbohydrate metabolism and are relevant for both veterinary and medical use.

## 2. Materials and Methods

### 2.1. Oregano and Feed Preparation

Dried Turkish oregano (Saucy Spice Company, NSW) was used to prepare the powder for the feed. The particle size of oregano with highest antimicrobial action against *Salmonella* was previously determined [17] and oregano powder was processed in a Planetary Ball Mill Machine (speed no. 5, 2 h, 40 g*each run; Changsha Yonglekang Equipment, China) and the dried powder passed through a 75 μm sieve. The particle size of the oregano powder used in feed, determined by laser diffraction (Mastersizer 2000, Malvern, ATA scientific, Taren Point, Australia) had an average diameter of 10 μm. 

Chicken diet formulated to meet or exceed the National Research Council guidelines for broiler chickens, commercially produced by Red Hen, (Laucke Mills, Australia). Red Hen broiler chicken ration is a blend of whole and rolled grains and micro pellets, certified drug free with no antimicrobials or coccidiostats. The feed contained the following ingredients: wheat, triticale, barley, oats, peas, lupins, lentils, beans, canola, soybean, sunflower, meatmeal, fish meal, blood meal, fat, molasses, limestone, di-calcium phosphate, sodium bicarbonate, potassium carbonate, salt, lysine, methionine, threonine, tryptophan. Vitamins supplemented include A, D3, E, K, thiamine (B1), riboflavin (B2), pantothenate (B5), pyridoxine (B6), B12, niacin, folic acid, biotin, and choline. Minerals supplemented in the ration were calcium, phosphorus, potassium, sodium, chloride, cobalt, copper, iron, iodine, manganese, molybdenum, selenium, and zinc. Final macronutrient content was 20% protein, 5% fat, 6.5% fiber, and 0.35% fat. The oregano was mixed into the feed at 2% (w/w).

### 2.2. Characterization of Oregano

The proportion of powdered oregano used as a feed additive in the trial was sent to ChemCentre analytical lab (Perth, Australia) operating as an Australian Government facility (www.chemcentre.wa.gov.au (accessed on 16 November 2020)). Total phenolics and terpenes were determined using the Folin–Ciocalteu method as described and analyzed on GS-MS (GC system—Agilent 7890A GC system connected to an Agilent 5977A MSD; Agilent Technologies, Santa Clara, CA, United States) using method described by Singleton et al. [18] and Phenomenex column—Zebron ZB-1MS, 60 m × 0.32 mm × 1.00 µm. Total dietary fiber was determined using the Megazyme total dietary fiber assay procedure as per the detailed protocol published by a supplier [19].

### 2.3. Birds and Management 

Day old Ross Broiler 308 chicks (Bond Enterprises, Toowoomba, Australia) were randomly distributed into two groups (*n* = 12 chicks/treatment) in temperature and light controlled room. All birds were fed ad libitum and had unrestricted access to drinking water. Birds were individually tagged using leg bands and weighed every week for a total of 42 days. Birds were euthanized at day 42 post-hatch (CO_2_, BOC, North Ryde, Australia) and dissected. Liver sections were taken from all birds for histology and RNA extraction. Four male birds were randomly selected from each treatment for RNAseq analysis. For each bird one cut was made from the same section of the liver and split into two cuts, one for histology and the other for RNAseq.

### 2.4. Histology

The tissue samples of liver were collected and fixed in 10% buffered formalin solution. Fixation, paraffin embedding, deparaffinization, rehydration, and staining with hematoxylin and eosin (H&E), were done by routine laboratory procedures. Glass slides were scanned at the Translational Research Institute Microscopy Core Facility (Brisbane, Australia) using a Nikon Brightfield, Olympus VS120 slide scanner (Olympus, Tokyo, Japan) and analyzed using Olympus microscopy software Olyvia (Olympus, Tokyo, Japan). 

### 2.5. RNAseq Sequencing 

About 100 mg of tissue samples were homogenized with 1 mL of TRIsure (Cat# BIO-38033, Bioline Meridian Bioscience, London, UK) using OMNI tissue homogenizer TH (OMNI International, Kennesaw, GA, USA) and centrifuged, followed by a phase separation adding 200 μL of chloroform and centrifugation. The RNA from the resulting aqueous phase was isolated and cleaned up using Bioline Isolate II RNA Mini Kit (Cat# BIO-52072) following the manufacturer’s instructions. Extracted RNA was sent to the sequencing facility in RNAstable tubes. The sequencing was performed in Macrogen, Inc., Korea. The library was prepared using TruSeq RNA Library Prep Kit v2 #RS-122-2001 (Illumina, San Diego, CA, United States), and sequenced on Novaseq 600 150 bp PE. Four samples were successfully extracted and sequenced from each control and oregano treatment liver with a total of eight sequenced samples. Sequencing data is available on the Sequence Read Archive (SRA) database (https://www.ncbi.nlm.nih.gov/sra (accessed on 16 November 2020).) under BioProject accession number PRJNA683135. 

### 2.6. Data Analysis, Software and Statistics

CLC Genomics Workbench by Qiagen Bioinformatics was used for RNAseq analysis. Quality trimmed sequences and detailed sequencing quality QC file is given in Table S1. Paired sequences were assigned to genes and transcripts against chicken genome (*Gallus_gallus*-5.0), using CLC Genomics Workbench recommended settings. Resulting raw matrix was used in DeSeq2 analysis to provide differential expression and fold change for each gene. DeSeq2 was done in R and the output was imported to Qiagen’s Ingenuity Pathway Analysis (IPA) software where the remaining analysis was performed using the IPA significance cut-offs as indicated in the results section. 

## 3. Results 

### 3.1. Animals and Trial

We translated the oregano concentration to phenolics and terpenes using chemical analysis. In dried powdered oregano, concentrations of 3-carene, alpha-pinene, alpha-terpinene, alpha-terpineol, beta-pinene, caryophyllene, citronellal, citronellol, eucalyptol, gamma-terpinene, geraniol, geraniol acetate, limonene, myrcene, p-cymene, terpine-4-ol, terpinolene were all under the detection limit. Linalool and thymol were both present at 0.01% (w/w), and, as expected, carvacrol dominated with 0.84% (w/w) of dried oregano. Total polyphenols in oregano were 37.8 g/kg of powdered oregano, making it 756 mg/kg in finished feed. Powdered oregano contained 52.6% total dietary fiber (final of 1.05% in finished feed). Thus final concentrations of oregano phytogenic products in chicken feed were 0.0168% carvacrol, and 0.0002% of thymol and linalool each. 

There was no difference in any of the health parameters (weight, mortality, feed intake) between oregano supplemented and control birds. This experiment focused on transcriptomics and molecular effects and was not investigating feed conversion ratio (FCR) or other performance measures to reduce the animal number as per the animal reduction ethics principles. The birds’ weights exceeded AVIAGEN recommended Ross Broiler 308 industry standard. More details on animal weights, feed consumption, microbiota composition, short-chain fatty acids, and multiple organ histology on this dataset can be found in Bauer at al. [17].

Comparison of liver histology between the control and oregano supplemented birds showed no visible changes in liver pathology or function (Figure S1). 

### 3.2. Sequencing Quality Control 

This dataset was sequenced remarkably deep, aiming to over 100 million paired sequences per sample, with a total number of paired, quality trimmed sequences for eight samples sequenced of 906,494,608 with average 113,311,826 paired and quality trimmed sequences per sample. Sequences were paired and quality trimmed to zero ambiguous, length of all sequences of 150–151 nt, and minimum PHRED score of 20, with 97.73% of sequences with PHRED> 30. A detailed sequencing QC file for each sample is given in Table S1.

### 3.3. Liver Gene Expression

There were 14,247 genes with transcripts detected as expressed in the liver in our study; Table S2 contains raw gene data matrix, as well as “Counts Per Million” (CPM), “Transcripts Per Million”(TPM) and “Reads Per Kilobase of transcripts per Million mapped reads” (RPKM) normalized data against the chicken genome annotations, while Table S3 provides DeSeq2 analysis outputs with log2 FC, P-values and FDR corrected statistics. Among the known and annotated genes most highly upregulated in the liver by oregano are *CACNG4*, *TEKT5*, *COL20A1*, *ALDH1L2*, *ADGRG7*, *OTOS*, *COL10A1* and *ANKRD22*, while the most downregulated known genes included *FGF19*, *RASGRF2*, *GUCY2C*, *RERG*, *MFAP3L*, *TACR1*, *CTSEAL*, *VTG2*, *CTNNA3*, *SMAD7B*, *UBAP2*, *SPIN1W*, *HNRNPKL*, and *NIPBLL*. 

### 3.4. Pathway Analysis

The Qiagen’s Ingenuity Pathway Analysis (IPA) analysis is based on the comprehensive, 9.6 million manually curated gene expression annotations, in the Ingenuity Knowledge Base (IKB) commercial database (Qiagen, 2020, Hilden, Germany). IPA uses IKB database to identify the most significant pathways affected by treatment and discover potential novel regulators, networks and connections associated with selected gene lists. IPA was able to map 11,101 out of 14,247 mapped chicken genes to IKB database, remaining genes were unknown and had no annotations. We performed IPA analysis of the genes differentially expressed with DeSeq2 *p* < 0.05 and with fold change cut-off between the groups of 1.2 or equivalent of a minimum 20% increase or decrease of DeSeq2 significantly differential genes.

**Figure 1:**
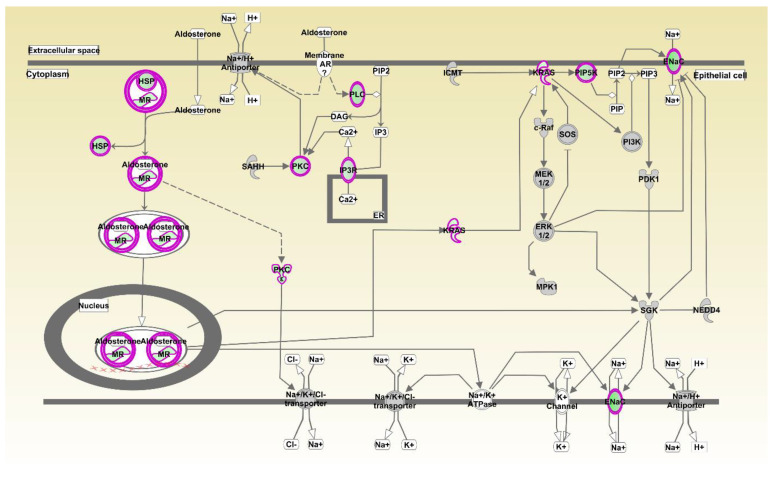
Aldosterone signaling pathway most significantly affected by oregano with 19 genes. Oregano significantly inhibited pathways included, IGF-1 Signaling, PI3K Signaling in B Lymphocytes, CREB Signaling in Neurons, Gαq Signaling, Thrombin Signaling, Production of Nitric Oxide and Reactive Oxygen Species in Macrophages, Synaptic Long Term Depression, IL-3 Signaling, LPS-stimulated MAPK Signaling, CCR3 Signaling in Eosinophils, α-Adrenergic Signaling, Growth Hormone Signaling, Melatonin Signaling, Pancreatic Adenocarcinoma Signaling, Apelin Endothelial Signaling Pathway, Th1 Pathway, IL-6 Signaling, with full pathway list and statistics provided in Table S4. Activated pathways were Colanic Acid Building Blocks Biosynthesis, BAG2 Signaling Pathway, LPS/IL-1 Mediated Inhibition of RXR Function, Antioxidant Action of Vitamin C, and PTEN Signaling, as in Table S4.

There were 33 pathways significantly inhibited and five pathways activated in the liver by oregano, as shown in Table S4. By far the most significant by the *p*-value and *z*-score was aldosterone signaling in epithelial cells (Figure 1), with 19 genes from this pathway among differentially expressed in liver between oregano and control samples: *HSP90AA1*, *HSP90AB1*, *HSPA2*, *HSPA5*, *HSPA8*, *HSPD1*, *HSPH1*, *DNAJA1*, *DNAJB5*, *DNAJC12*, *DNAJC6*, *ITPR3*, *KRAS*, *NR3C2*, *PIKFYVE*, *PLCB1*, *PRKCE*, *PRKCI*, and *SCNN1B*. Please note that IPA pathway (Figure 1) does not show or color the expression of all individual genes involved in the pathway, but rather it colors inhibited arms of the pathway green and activated as red. Inhibition, in this case (Figure 1), can result from upregulation of genes that inhibit or from downregulation of the genes that enhance the pathway. 

### 3.5. Upstream Regulators 

We proceeded with the IPA Upstream Analysis to predict upstream transcriptional regulators that can explain the observed gene expression in the liver between oregano supplemented and control fed birds. IPA’s definition of the upstream transcriptional regulator is extensive and includes any molecule that can affect gene expression, from transcription factors to micro RNA, enzymes, chemicals, or registered drugs. It is important to note that the database will report any compound including other species transcription factors, if there is evidence in IKB dataset that they could regulate the genes from our submitted query list. For that reason, Table S5 provides full data, including a fold change of transcription factors to indicate their presence/absence in our dataset. 

IPA analysis predicted 19 molecules or other complex products that, based on literature annotated in IKB database, can regulate differential genes in the same way as oregano and thus get their effects amplified if used or, in case of transcription factors, activated together. They included drugs geldanamycin, cephaloridine and tanespimycin; two toxic chemicals 2-amino-1-methyl-6-phenylimidazo-4-5-b-pyridine, and 6-hydroxydopamine; CD3 complex, enzymes KRAS, and KDM1A; G-protein coupled receptor CNR1; MTOR kinase, three micro RNAs miR-34a-5p (and other miRNAs w/seed GGCAGUG), miR-1-3p (and other miRNAs w/seed GGAAUGU) and mir-210; five transcription regulators HSF1, RUNX1, STAT6, PML, PAX5, and a transmembrane receptor TLR9 (noting that in chicken TLR21 acts as a functional homologue to mammalian TLR9, and would be activated instead). Out of the regulators above, *MTOR*, *KRAS*, *PML*, *PAX5*, and *KDM1A* were expressed in our dataset. 

The analysis reported 23 predicted inhibited regulators, molecules that, based on existing literature, can regulate genes in the opposite way to oregano, thus inhibiting each-others transcriptional action: two endogenous chemicals aldosterone and dihydrotestosterone; five chemical drugs BMS-690514, sirolimus, metformin, resveratrol, and cocaine; two chemicals SAHM1 and forskolin, genes encoding different cellular proteins TRAP1, NR3C1, CD24, PTEN, SGPP2; transcription regulators HDAC5, NEUROG3, GLI1, SIM1, HNF4A, EP300, ARNT2, FOXO3, and a transmembrane receptor CAV1.

**Figure 2:**
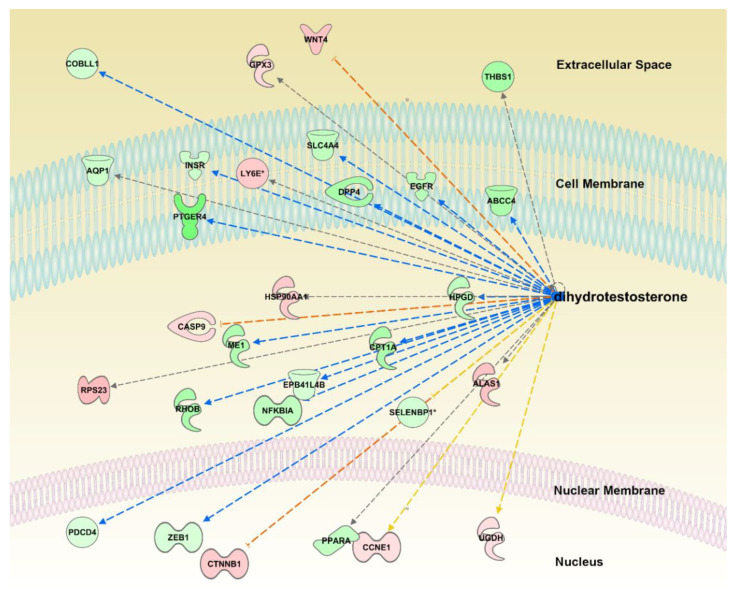
Predicted inhibition of the dihydrotestosterone (DHT) transcriptional regulation. Yellow lines indicate that the direction of gene expression does not agree with predicted inhibition; grey line indicates inconsistent literature on activation/inhibition of the gene by the regulator. Blue line indicates that if the DHT is inhibited the gene should be downregulated (green). Red line indicates that if DHT is inhibited the gene is expected to be upregulated (red).

The most inhibited upstream regulator was dihydrotestosterone *(p =* 8.95 × 10^−5^, *z-score* = −3.162, Figure 2). There were 29 genes regulated by dihydrotestosterone among our differential genes, 26 were expressed consistently with inhibition of dihydrotestosterone (opposite to the action of dihydrotestosterone; Figure 2). All predicted upstream regulators, with their fold change in our dataset, corresponding IPA statistics and target genes from our differential DeSeq2 list, are given in Table S5.

**Figure 3:**
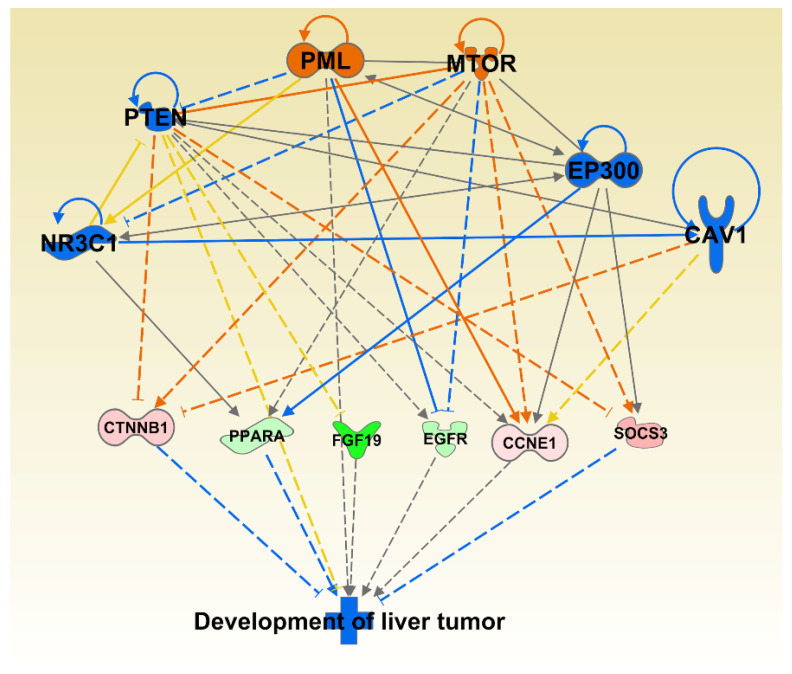
Multiple significantly affected transcriptional regulators acting towards inhibiting the liver tumor category. The shape of the molecules is indicting molecular function as per legend given in Figure S2. The genes are colored by the level of expression in shades of red (upregulated) or green (downregulated) while the regulators are colored blue for inhibited or orange for activated function. Red lines indicate activation, blue lines inhibition, grey lines inconsistent findings, and yellow lines indicate that the state of the gene is opposite from the literature based prediction.

We also investigated synergistic regulator effects looking for multiple regulators that are either activated or inhibited to potentially regulate diseases or functions synergistically. The upstream regulators CAV1, EP300, NR3C1, PTEN, MTOR, and PML were expressed in our dataset, activated or inhibited in the direction that could lead to inhibition of Development of Liver Tumor disease category, one of the significantly affected disease categories; *p =* 5.55 × 10^−6^ (Figure 3). 

### 3.6. Diseases and Functions

**Figure 4:**
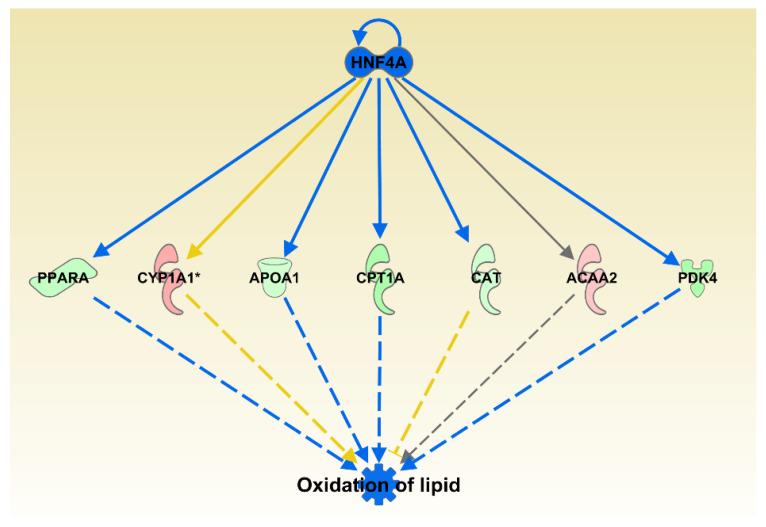
Oxidation of lipid was one of the IPA predicted functions inhibited by oregano with 16 genes from the category differentially expressed. The genes are colored by the level of expression in shades of red (upregulated) or green (downregulated) while the regulators and functions are colored blue for inhibition. Red lines indicate activation, blue lines inhibition, grey lines inconsistent findings, and yellow line indicates that the state of the gene is opposite from the literature based prediction.

We next used IPA’s Diseases and Functions analysis that is comparable to typical gene ontology analysis. This analysis compares the query list of differentially expressed genes with the genes associated with specific diseases and functions based on annotations from IKB database. There were three categories that were significantly increased: Peripheral Vascular Disease, Urination Disorder and Cerebrovascular Dysfunction; 12 significantly inhibited functions and diseases: Incidence of Tumor, Frequency of Tumor, Tumorigenesis of Epithelial Neoplasm, Development of Malignant Tumor, Development of Carcinoma, Development of Digestive Organ Tumor, Transport of Molecule, Development of Liver Tumor, Cell Viability of Breast Cancer Cell Lines, Invasion of Epithelial Cell Lines, Quantity of Neurons, and Oxidation of Lipid (Figure 4). 

Separately, the upstream analysis identified one transcription regulator, Hepatocyte Nuclear Factor 4 alpha, HNF4A, that was expressed as downregulated in our dataset and identified as an inhibited transcription factor capable of affecting 7 of the 16 genes from the Oxidation of Lipid category. Deeper inspection of all significantly altered subcategories from Lipid Metabolism category indicated that highest contributor to overall category inhibition comes from inhibition of Quantity of Steroid Hormone (*p* = 6.07 × 10^−6^, *z* = −1.92) and Fatty Acid Metabolism (*p* = 1.9 × 10^−4^, *z* = −1.39). 

There was a range of significantly affected diseases by *p*-value, with activation/inhibition based on *z*-score that can be observed on the Diseases and Functions heatmap in Figure S3 and the full list of diseases and functions with *p*-values and *z*-scores given in Table S6.

There were 281 cancer-related categories significantly altered by oregano with zero categories with *z*-score higher than 1 that would indicate disease activation, and 33 cancer categories with *z*-score lower than −1 indicating disease inhibition, and a *p*-value ranging from 4.6 × 10^−4^ to 1.4 × 10^−17^. Those inhibited cancer disease categories include Development of Liver Tumor, Tumorigenesis of Epithelial Neoplasm, Development of Digestive Organ Tumor, Development of Hepatocellular Carcinoma, Head and Neck Cancer, Skin Cancer, B Cell Cancer, Development of Lung Tumor, Abdominal Neoplasm, Lymphoreticular Neoplasm, Urinary Tract Tumor, and others listed in Table S6.

**Figure 5:**
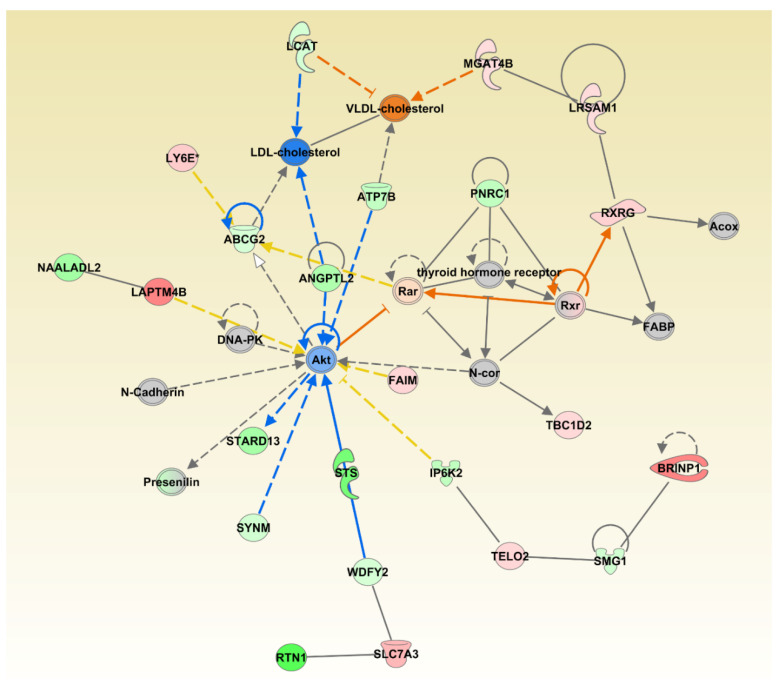
One of the top-scoring most affected network interactions included genes from the Carbohydrate Metabolism and Lipid Metabolism categories. The shape of the molecules is indicting molecular function as per legend given in Figure S2. Yellow lines indicate that the direction of gene expression does not agree with literature; grey line indicates inconsistent literature on activation/inhibition of the gene by the regulator. Blue line indicates expected inhibition and red line indicates expected activation.

Among the functions significantly affected by oregano supplementation, were eight categories from carbohydrate and 13 from lipid metabolism all highly significantly altered (*p* = 7.67 × 10^−4^ to 1.26 × 10^−8^). Based on *z*-scores, functions inhibited by oregano were Metabolism of Carbohydrate, Synthesis of Carbohydrate, Uptake of Monosaccharide, Glycolysis of Tumor Cell Lines, Uptake of 2-Deoxyglucose, and Uptake of D-glucose in Carbohydrate Metabolism category. In Lipid Metabolism category inhibited functions were Quantity of Steroid Hormone, Fatty Acid Metabolism, Quantity of Steroid, Quantity of Polyunsaturated Fatty Acids, Conversion of Lipid, Metabolism of Acylglycerol, and Concentration of Lipid (Table S6). It should be noted that there is a strong overlap of genes between the lipid and carbohydrate metabolism categories. Figure 5 shows an overlapping network of interactions between carbohydrate and lipid metabolism. 

Accumulatively, the number of the significantly affected and slightly activated or inhibited diseases, taken together, give a clear pattern of the oregano influence. Toxicology functional analysis in IPA inspected organ toxicity of oregano supplementation (Table S7). Based on liver gene expression, Development of Liver Tumor category, was significantly (*p* = 3.25 × 10^−5^) decreased (*z* = −2.39), with 13 genes altered in the direction opposite from liver tumor development pattern.

## 4. Discussion

**Figure 6:**
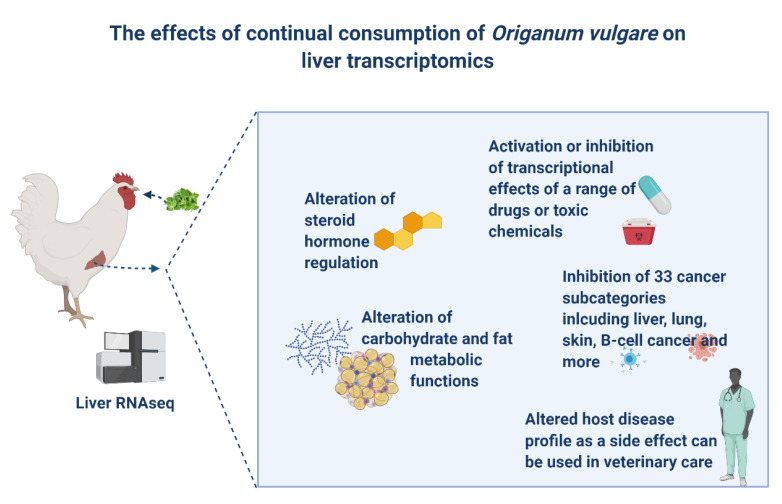
An overview of the main effects of prolonged oregano consumption on liver transcriptomics.

Oregano is one of the most commonly used natural products in poultry production. Despite its “natural” status, our results have shown that oregano is a complex compound with diverse biological effects with implications not only in poultry but also in human health. Continual prolonged consumption of oregano resulted in a range of changes to the liver gene expression in broilers with most prominent effects being inhibition of steroid hormones, fat and carbohydrate metabolism, and a number of cancer disease categories (Figure 6). 

Aldosterone Signaling in Epithelial Cells is the most inhibited pathway in the liver of the birds fed oregano continuously for 6 weeks. Aldosterone is the primary mineralocorticoid steroid hormone produced by the adrenal gland. It is essential for sodium conservation in the kidney, salivary glands, sweat glands, and in the colon and plays a central role in the regulation of blood pressure, sodium and potassium levels [20]. Aldosterone production and signaling are disrupted in obesity [21]. Aldosterone dysregulation is pathologic and linked to cardiovascular and kidney disease. Low levels of aldosterone result in lowered blood pressure, an increase of potassium levels and lethargy, however, it should be noted that inhibited aldosterone signaling does not necessarily mean reduced aldosterone production or circulating blood levels. A major mode of action of aldosterone is via modifying gene expression [20] by repressing and inducing many genes. 

In the liver, the aldosterone level is increased in liver fibrosis [22] and can increase the levels of liver fat consistently enough to be used as a predictor of liver fat levels [23]. Aldosterone is also involved in the pathogenesis of the fatty liver disease [24] and in the pathogenesis of obesity and overweight [25,26]. Fat metabolism and oxidation were also inhibited in our dataset (Figure 4) and reduction of the fat oxidation category is likely partially controlled by the inhibited and downregulated HNF4A—hepatocyte nuclear factor 4 alpha transcription factor (Figure 4). This is consistent with findings of Sabino et al. [13] who also investigated liver transcriptomics in broiler chickens fed oregano extract. They also reported significant downregulation of genes involved in fatty acid metabolism in addition to the insulin signaling pathway, which was not observed in our study. Whether the transcriptional reduction of aldosterone signaling pathway has any connection with reduced levels of aldosterone and resulting actual liver fat levels is still to be established. The overlap of genes between the fat metabolism subcategories should be noted, and this effect on liver fat metabolism could be largely due to effects on steroid arm of fat metabolism.

There was a range of significantly inhibited pathways that are related to aldosterone signaling: Renin-Angiotensin Signaling, α-Adrenergic Signaling, and Growth Hormone Signaling. In addition to altered pathways, aldosterone as well as dihydrotestosterone were among the inhibited transcriptional regulators, i.e., oregano supplementation significantly reversed their gene expression effects. Dihydrotestosterone is an androgen hormone, made through the conversion of testosterone [27]. Almost 10% of the testosterone produced by an adult person is converted in the testes and prostate, or in the ovaries in women, to dihydrotestosterone [27]. Dihydrotestosterone is much more potent than testosterone, and many of the testosterone actions occur after it is converted to dihydrotestosterone.

We have previously reported functional analysis of the microbiota genetic potential [28] showing that 2% oregano in feed significantly reduced the abundance of intestinal bacterial genes involved in steroid hormone biosynthesis via Picrust functional prediction [28], we confirmed this with ileum transcriptomics, and now also in the liver. Others have shown that oregano can induce estrogenic responses in vitro and can also show estrogen-like activity [29], thus influencing steroid hormone balance. This may be beneficial as the means to help regulate steroid hormone-based imbalances, especially if the effects could be pinned to individual components of oregano which is necessary for reproducibility of the outcome.

In addition to oregano’s effect of key metabolic and steroidal pathways, our data also suggest that it may also influence the functioning of several commercially available drugs or supplements. Forskolin, listed in IKB as a chemical toxicant is produced by the roots of the Indian plant *Coleus forskohlii* and has been used for centuries in traditional medicine, with safety recognized in modern medicine. Forskolin directly activates the adenylate cyclase enzyme, that generates cAMP from ATP, and consequently, raising intracellular cAMP levels [30]. This feature of forskolin is beneficial against cancers [31,32] and recently synthesis of novel forskolin derivates for anticancer drug development and suggestions for higher use as an anticancer drug ensued [33,34]. Forskolin is marketed as a weight loss pill, also backed up by research [35]. In the liver, forskolin can significantly diminish liver fibrosis acting as an antioxidant and anti-inflammatory agent via inhibition of hedgehog signaling, mediated by cAMP-dependent activation of PKA [36]. Oregano significantly changed the expression of 31 genes from the group of genes regulated by forskolin, with only 3 of the 31 genes inconsistent with the inhibition (reversal) of forskolin transcriptional profile. 

Prolonged use of oregano also inhibited transcriptional pathways mediated by Sirolimus, an anticancer agent and immunosuppressant mTOR inhibitor, marketed as Rapamune and Rapamycin. It is used to coat coronary stents [37], prevent organ transplant rejection, especially for kidney transplantation [38,39] and treat vascular anomalies [40] and a rare lung disease called lymphangioleiomyomatosis [41]. IKB database lists 1508 clinical trials investigating the role of Sirolimus in 424 medical conditions including a range of cancers and immunological conditions as well as Crohn’s disease, range of kidney disorders, liver disease, coronary disease, atherosclerosis, and more. 

Another drug with alleged anticancer action whose effects on gene expression are significantly reversed by oregano is resveratrol, a natural phenol produced by some plants in response to injury or infection. Resveratrol is commonly used as a dietary supplement and studied as a potential treatment in many human diseases. IKB reports 70 clinical trials on 40 conditions including polycystic ovary syndrome, knee osteoarthritis, infertility condition, insulin resistance, schizophrenia, metabolic syndrome X, spastic paraplegia, diabetes mellitus, Alzheimer’s disease, Friedreich ataxia, Parkinson’s disease, memory, stage III colon cancer, stage II colon cancer, colon cancer, cancer, rectum cancer, colon adenocarcinoma, colorectal tumor, adult solid tumor, hepatitis B, vascular injury, hepatitis, ophthalmic disorder, disorder of lipid metabolism, and others. 

The effects of resveratrol on cancer have been extensively studied, but they gave highly inconsistent findings even at high doses used [42]; similarly, there is contradicting evidence on its role in diabetes, cardiovascular health, and neurological disease [43]. However, there is evidence that resveratrol improves lifespan and reduces aging [44] and can limit secondary damage after ischemic stroke or acute brain trauma [45]. 

Oregano also significantly reversed the transcriptional effects of the anticancer drug BMS-690514 (a VEGFR and EGFR tyrosine kinase inhibitor), cocaine, and also of metformin, a drug used for the treatment of type 2 diabetes and of polycystic ovary syndrome. Possible interference of prolonged oregano use on the range of drugs that have such wide effects on the diseases and physiological functions makes the predictions of oregano enabled final modifications of the liver function very challenging. Based on ileum gene expression oregano also inhibited the effects of the contraceptive pill. At the same time, oregano may amplify the effects of the geldanamycin, antitumor, antibiotic, and geldanamycin and its derivate tanespimycin, as well as a semisynthetic derivative of cephalosporin C named Cephaloridine and changes the expression and regulation of genes involved in a range of diseases. 

The upstream regulators CAV1, EP300, NR3C1, PTEN, MTOR, and PML were expressed in the direction that could lead to inhibition of Development of Liver Tumor category. While this combination of genes may lead to the inhibition of liver cancer development, changes to individual genes, such as the inhibition of the tumor suppressor gene Phosphatase and tensin homologue deleted on chromosome 10 (PTEN) may have the potential to increase the development of other neoplasms. PTEN acts on the phosphoinositide 3-kinase (PI3K)-AKT-mammalian target of rapamycin (mTOR) pathway and is one of the most commonly disrupted tumor suppressors in human cancer [46]. Its inhibition or loss is considered a founder genetic event for the induction and progression of tumor development [47]. Natural products that have the potential to affect this important tumor suppressor gene should be further evaluated. 

We have recently reported the transcriptomic changes in the ileum of the same birds [48]. While the effects on steroid hormones and rearrangement of cancer category were consistent, in the ileum, we noted the inhibition of brain–gut axes signaling, a strong reduction of goblet cells, and intestinal inflammation in oregano supplemented birds. This suggests the importance of transcriptomic evaluation of multiple organs to elucidate the effects of nutritional supplements in nutrigenomic studies. 

Despite the inhibition of transcriptional effects of many antitumor drugs and supplements and transcriptional regulators, our disease and function analysis suggest that oregano use may also lead to inhibition of the range of tumors including liver tumor and to possible enhancement of peripheral vascular disease, urination disorder, and cerebrovascular dysfunction. 

## 5. Conclusions

The results of this study have pointed to the biologically diverse effects of oregano on gene expression, outside of its intended antimicrobial purpose. Whilst most of the affected functions have little or no relevance for the use of phytogenic products in the poultry industry, as chickens do not take any of the affected drugs and, at least during the production lifespan, are not bothered with cancer or hormonal imbalance, it does highlight potential unintended consequences of the prolonged use of natural products and may have broader implications in both animal and human health. To eventually exploit the effects of oregano for clinical use in both supplemented livestock and humans, individual compounds need to be investigated to isolate those responsible for the clinically useful features of oregano and potential side-effects evaluated. 

## Data Availability

Publicly available datasets were analyzed in this study. This data can be found in Sequence Read Archive (SRA) database: [(https://www.ncbi.nlm.nih.gov/sra (accessed on 16 November 2020)) under BioProject accession number PRJNA683135].

## Supplementary Materials

The following are available online at https://www.mdpi.com/2076-2615/11/2/398/s1, Figure S1: Histology, Figure S2: Legend, Figure S3: Diseases and functions heatmap, Table S1: QC report, Table S2: Expression matrix, Table S3: DeSeq2 outputs, Table S4: Pathways, Table S5: Upstream regulators, Table S6: Diseases and functions, Table S7: Toxicology.

## Abbreviations

EO—essential oil; AGP—antibiotic growth promoter; NE—necrotic enteritis; AMR—antimicrobial resistance; WHO—World Health Organization.
